# Overexpression of Circ-Astn1 Suppresses Hyperglycemia-Induced Endothelial Cell Damage via the miR-138-5p/SIRT1 Axis

**DOI:** 10.1007/s11596-025-00011-9

**Published:** 2025-02-27

**Authors:** Hong-bin Yu, Li-yun Wang, Xiao-ning Yan, Xue-yan Wu, Jian-long Wu, Da-wei Liu, Si-yang Liu

**Affiliations:** 1 Chengde Central Hospital, Chengde, 067000 China; 2 Shanxi Hospital of Integrated Traditional and Western Medicine, The Fourth Clinical College, Shanxi University of Chinese Medicine, Jinzhong, 030619 China; 3 Department of Human Anatomy, Chengde Medical College, Chengde, 067000 China

**Keywords:** Circ-Astn1, Endothelial cell, Hyperglycemia, miR-138-5p, SIRT1

## Abstract

**Objective:**

To elucidate the regulatory mechanism of circRNAs in diabetic retinopathy.

**Methods:**

Next-generation sequencing (NGS) was employed to identify circRNAs that are abnormally expressed in endothelial progenitor cells (EPCs) under hyperglycemia (HG) conditions. The regulatory mechanism and predicted targets of this circRNA were also studied via bioinformatics analysis, luciferase reporter assays, angiogenic differentiation experiments, flow cytometry, and RT-qPCR.

**Results:**

Circ-astrotactin 1 (circ-Astn1) expression was decreased in EPCs under HG conditions, and circ-Astn1 overexpression inhibited HG-induced endothelial damage. The miR-138-5p and silencing information regulator 2 related enzyme 1 (SIRT1) were identified as circ-Astn1 downstream targets, which were further verified through luciferase reporter assays. SIRT1 silencing or miR-138-5p overexpression reversed the protective effect of circ-Astn1 on HG-induced endothelial cell dysfunction, as evidenced by increased apoptosis, abnormal vascular differentiation, and inflammatory factor secretion. SIRT1 overexpression reversed miR-138-5p-induced endothelial cell dysfunction under HG conditions. In vivo experiments confirmed that circ-Astn1 overexpression promoted skin wound healing through the regulation of SIRT1.

**Conclusions:**

These findings suggest that circ-Astn1 promotes SIRT1 expression by sponging miR-138-5p. Circ-Astn1 overexpression suppresses HG-induced endothelial cell damage via miR-138-5p/SIRT1 axis.

## Introduction

Diabetes mellitus (DM) continues to become increasingly burdensome to the global healthcare system [1, 2]. Diabetes is correlated with impaired wound healing, increasing susceptibility to chronic wounds that do not heal [3]. The wounds precede 84% of all diabetic lower extremity amputations, and patients have a 50% 5-year mortality rate after amputation [4]. Chronic diabetic wounds display a persistent inflammatory state with elevated protease and proinflammatory cytokine levels, as well as vascular endothelial cell impairments [5]. Endothelial cells are the first obstacle between the blood and the vascular wall. Additionally, the endothelial glycocalyx is made of glycosaminoglycans, proteoglycans and glycoproteins, which coat endothelial cell surfaces and take part in vascular homeostasis maintenance. More evidence indicates that endothelial dysfunction, such as decreased nitric oxide (NO) levels and increased endothelial permeability, is induced by hyperglycemia (HG), which usually occurs in the blood vessels of diabetic patients [6]. However, the precise mechanism of vascular endothelial injury in diabetes is unknown.

Noncoding RNAs (ncRNAs) constitute the majority of cellular RNAs, accounting for > 90% of human RNAs [7–9]. Research has shown that ncRNAs are as pivotal as proteins and act as underlying players in multiple cellular procedures, such as cell apoptosis, immune response, migration, angiogenesis and proliferation [10–12]. Circular RNAs (circRNAs) are regarded as critical human disease regulators and are used as biological markers in some diseases, including diabetes [13, 14]. Competing endogenous RNA (ceRNA) function is considered a crucial mechanism by which circRNAs regulate gene expression, and ceRNAs can also be used as disease biomarkers. Theoretically, circRNAs act as molecular sponges of microRNAs (miRNAs), which bind to miRNA response elements within circRNAs, resulting in suppressed expression and all target gene functions related to the respective miRNA family [15].

Previous studies have shown that circRNA_0084043 contributes to diabetic retinopathy progression through miR-140-3p sponging and the induction of transforming growth factor alpha (TGFα) gene expression in retinal pigment epithelial cells [16], and silencing circ-ZNF609 ameliorates vascular endothelial dysfunction [17]. However, the regulatory role of circRNAs in HG-induced endothelial dysfunction remains largely unknown. In this context, our current work aims to illuminate the regulatory mechanism of circRNAs in diabetic retinopathy. These results show that circ-astrotactine 1 (circ-Astn1) promotes silencing information regulator 2 related enzyme 1  (SIRT1) expression through the sponging of miR-138-5p. Circ-Astn1 overexpression suppresses HG-induced endothelial cell damage via miR-138-5p/SIRT1 axis.

## Materials and Methods

### Diabetic Wound Induction

Diabetes was induced in 18 BALB/c mice via the intraperitoneal injection of 60 mg/kg single dose streptozotocin (STZ) dissolved in 0.1 mmol/L citrate buffer (pH = 4.5). Three days after STZ administration, diabetes was confirmed by measurement of fasting blood glucose levels after blood was extracted from the tail vein. Mice with fasting blood glucose levels > 250 mg/dL were regarded as diabetic. We maintained the mice for an additional month, at which time they were used for experiments after blood glucose was stabilized. Following diabetes validation, the mice were anaesthetized via intramuscular injection of xylazine cocktail and ketamine hydrochloride at 10 mg/kg and 80 mg/kg, respectively. The hair in the dorsal leg area, which was sterilized with povidone iodine solution, was removed by a technician. A sterile biopsy punch was employed to make a full-thickness excisional 4 mm wound. Each mouse received a 5 mg/kg/day dose of the SIRT1 inhibitor EX-527 (Selleck, S1541, USA) or a local injection at the injury site with a circ-Astn1-overexpressing lentiviral vector. After 4 weeks, the mice were euthanized, and skin specimens were harvested for histopathological analysis.

C57BL mice were housed under controlled conditions (22 ± 2 °C, 60% ± 5% relative humidity and a 12 h light/dark cycle). Technicians performed the experiments following ethical animal research guidelines, which were monitored by the Institutional Animal Care and Use Committee in Chengde Central Hospital.

### Immunohistochemical Analysis

The tissue samples were fixed with 4% paraformaldehyde solution and then embedded in paraffin. The sections were sectioned and cultured overnight with primary antibodies against CD31 overnight at 4 °C and then with secondary antibodies (Abcam, UK) for 1 h at 37 °C. The sections were stained with 3,3ʹ-diaminobenzidine and counterstained with hematoxylin.

### Cell Culture

The endothelial progenitor cell (EPC) line was purchased from ATCC (Rockville, USA). We cultivated the cells in DMEM supplemented with 10% FBS (Gibco, USA) with the antibiotic streptomycin/penicillin G (Sigma-Aldrich, USA) for regular culture. For HG induction, we cultured EPCs in HG medium containing 30 mmol/L glucose for 48 h.

Our team obtained HEK293 cells from ATCC, and stored them in DMEM containing 10% FBS at 37 °C in an atmosphere with 5% CO_2_.

### High-throughput and Strand-specific RNA-Seq Library

Total RNA was extracted from the serum of diabetic and control mice with TRIzol Reagent (Invitrogen, USA). For each sample, 3 μg of total RNA was subjected to VAHTS Total RNA-seq (H/M/R) Library Prep Kit from Illumina (Vazyme Biotech Co., Ltd., China) to eliminate ribosomal RNA and retain the remaining RNA, such as mRNAs and ncRNAs. Our team treated purified RNA with RNase R (Epicenter, 40 U, 37 °C for 3 h), followed by TRIzol purification. Our laboratory generated an RNA-seq library via a KAPA Stranded RNA-Seq Lib Prep Kit (Roche, Switzerland), and we subjected to next-generation sequencing (NGS) with an Illumina HiSeq 4000 (Aksomics, Inc., China).

### Enzyme-linked Immunosorbent Assay (ELISA)

Our laboratory assessed interleukin-6 (IL-6), tumor necrosis factor (TNF)-α and IL-1β in EPC supernatant via commercially available ELISA kits (BD Biosciences, USA) following a standard protocol.

### Flow Cytometric Analysis of Apoptosis

EPC apoptosis was measured via flow cytometry after the cells were labelled with propidium iodide (PI) and anti-annexin V (eBioscience, USA).

### Tubule Formation Assay

Technicians have assayed in vitro neovascularization by applying a human fibrin matrix. Following treatment, we seeded serum-starved EPCs in endothelial basal medium at 10^5^ cells per well in 6-well plates, which were coated with Matrigel (BD Biosciences, USA) and incubated at 37°C for 0.5 days. Our team observed tubular structures made in Matrigel and photographed them via phase-contrast microscopy. We measured newly formed tube lengths in 10 randomly generated fields per well.

### Bioinformatics Analysis

The predicted interactions between circRNAs, miRNAs, and mRNAs were identified via the StarBase database (http://starbase.sysu.edu.cn/).

### RNA Interference or Overexpression

The miR-138-5p mimics, the circ-Astn1 overexpression vector (circ-Astn1), the SIRT1 silencing vector (siSIRT1), the miR-138-5p suppressor, and the SIRT1 overexpression vector (SIRT1) were purchased from RiboBio (China). The cells were transfected via Lipofectamine 2000 (Thermo Fisher Scientific, USA).

### Quantitative Real-time Polymerase Chain Reaction (qPCR)

Our lab extracted total RNA from wound skin tissue or cells via a TRIzol reagent kit (Invitrogen, USA). Our team synthesized cDNA that was amplified via a TaqMan miRNA Reverse Transcription Kit. Our team performed qPCR with a TaqMan™ MicroRNA Assay Kit (#4,440,885, Applied Biosystems, USA), and the 2^−ΔΔCT^ method was used to measure relative expression fold changes. The technician utilized *U6* along with glyceraldehyde 3-phosphate dehydrogenase (*GAPDH*) as an internal reference. We employed the following primers: circ-*Astn1* forward, 5ʹ-CTCCTGGACCCTTGTGAAC-3ʹ and reverse, 5ʹ-CACCAGCGCCTGCGAGTGTAC-3ʹ; miR-138-5p, 5ʹ-AGCTGGTGTTGTGAATCAGGCCG-3ʹ; *SIRT1* forward, 5ʹ-TGATTGGCACCGATCCTCG-3ʹ and reverse, 5ʹ-CCACAGCGTCATATCATCCAG-3ʹ; *U6* forward, 5ʹ-CTCGCTTCGGCAGCACA-3ʹ and reverse, 5ʹ-AACGCTTCATTTGCGT-3ʹ; and *GAPDH* forward, 5ʹ-AATCCCATCACCATCTTCC-3ʹ and reverse, 5ʹ-CATCACGCCACAGTTTCC-3ʹ.

### Cell Apoptosis Assay

Our team added all of flow mix to the binding buffer after the harvested cells were washed twice with ice-cold PBS. A mixture including 5 μL of Annexin V/FITC and an equivalent volume of PI (BD, USA) was used to stain the cells for 0.25 h in the dark. Then, 400 μL of binding buffer was added. A FACSCalibur flow cytometer (BD Biosciences, USA) was used to analyze cellular apoptosis.

### Dual-Luciferase Reporter Assay

Our team cloned the putative miR-138-5p binding site into the 3ʹ-UTR of the target gene SIRT1 and circ-Astn1 mutant (MUT) or wild-type (WT) into the psi-CHECK vector (Promega, USA) downstream of the firefly luciferase 3ʹ UTR or circ-Astn1 as the primary luciferase signal. The vectors used were SIRT1-WT/circ-Astn1-WT and SIRT1-MUT/circ-Astn1-MUT. Renilla luciferase was used as a normalization signal. The psi-CHECK vector yielded a Renilla luciferase normalization signal to compensate for alternations in transfection and harvesting efficiency. Our team transfected HEK293 cells with Lipofectamine 2000 (Invitrogen Life Technologies, USA). Our team captured Renilla and firefly luciferase activities 1 day after transfection with a Dual-Luciferase Reporter Assay System (Promega, Germany) via a luminometer (Molecular Devices, USA). The relative Renilla luciferase activity was analyzed following the manufacturer’s protocols.

### Statistical Analysis

Our team represented the data as the mean ± SD. A statistical researcher used GraphPad Prism (GraphPad, USA) to calculate differences between groups. *P-*values ≤ 0.05 were considered statistically significant.

## Results

### Circ-Astn1 Functions Importantly in HG-induced Endothelial Cell Dysfunction

**Fig. 1:**
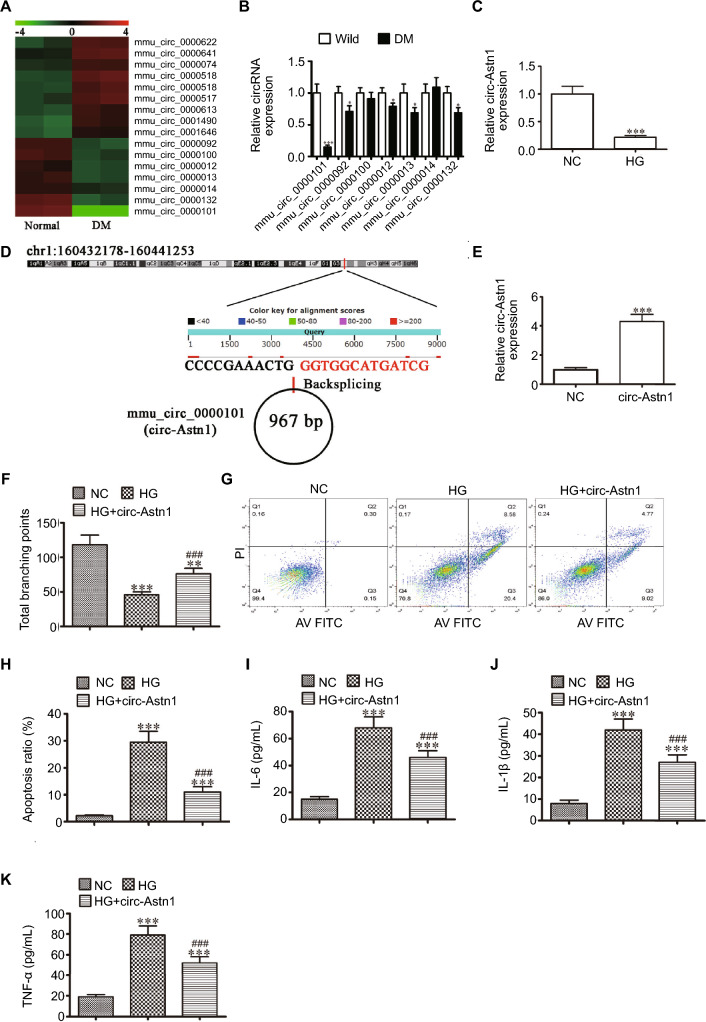
The important role of circ-Astn1 in hyperglycemia (HG)-induced endothelial cell dysfunction. **A** Heatmap showing serum circRNA expression in diabetic mice. **B** RT-qPCR analysis revealing the 8 downregulated circRNAs in serum of diabetic mice and wild-type (WT) mice. The data are expressed as mean ± SD. ^*^P < 0.05, ^***^P < 0.001 *vs*. WT mice. **C** RT-qPCR analysis showing circ-Astn1 expression in endothelial progenitor cells (EPCs) under normal conditions (NC, 5.5 mmol/L glucose) or HG conditions (30 mmol/L glucose). The data are expressed as the mean ± SD. ^***^P < 0.001. **D** Chromosomal localization of mmu_circ_0000101 (circ-Astn1). **E** RT-qPCR analysis of circ-Astn1 expression in EPCs transfected with circ-Astn1 overexpression vectors (circ-Astn1) or a negative control (NC). The data are expressed as the mean ± SD. ^***^P < 0.001. **F** The tube formation ability of EPCs. The data are expressed as the mean ± SD. ^**^P < 0.01, ^***^P < 0.001 *vs*. NC; ^###^*P* < 0.001 *vs*. HG. **G** and **H** The percentage of apoptotic EPCs determined by annexin-V/PI staining after 1 day under NC or HG conditions. The data are expressed as the mean ± SD. ^***^P < 0.001 *vs*. NG, ^###^*P* < 0.001 *vs*. HG. PI: propidium iodide. **I–K** The levels of the inflammatory cytokines IL-6 (**I**), IL-1β (**J**), and TNF-α (**K**) measured via ELISA. The data are expressed as the mean ± SD. ^***^P < 0.001 *vs*. NC, ^###^*P* < 0.001 *vs.* HG

Previous investigations revealed that ncRNAs play pivotal roles in diabetes pathogenesis [18]. CircRNAs belong to the family of endogenous noncoding RNAs, and abnormal circRNA expression can lead to vascular dysfunction in DM [19]. The present study used NGS to characterize circRNAs that are expressed in the serum of control and diabetic mice. We identified several abnormally expressed circRNAs (Fig. 1A). RT-qPCR analysis of 7 downregulated circRNAs (mmu_circ_0000101, mmu_circ_0000092, mmu_circ_0000100, mmu_circ_0000012, mmu_circ_0000013, mmu_circ_0000014, and mmu_circ_0000132) from the NGS results. The results revealed that only mmu_circ_0000101 was downregulated significantly in diabetic mouse serum (Fig. 1B), suggesting that mmu_circ_0000101 plays a regulatory role in DM development. RT-qPCR analysis revealed that mmu_circ_0000101 expression decreased in EPCs under HG conditions (Fig. 1C). Using bioinformatics analysis (http://www.circbase.org/), we found that mmu_circ_0000101 was 967 bp in length and located within the *Astn1* gene at chr1:160,432,178–160441253. Thus, mmu_circ_0000101 is referred to as circ-*Astn1* (Fig. 1D). Next, we constructed a circ-Astn1 overexpression vector and transfected it into EPCs. RT-qPCR was used to measure circ-Astn1 expression, and our laboratory revealed that circ-Astn1 expression increased significantly following transfection with the circ-Astn1 overexpression vector compared with the negative control (NC; Fig. 1E). Angiogenic EPC differentiation revealed that, compared with the negative control conditions, HG conditions significantly decreased the amount of capillary-like tube structures. However, circ-Astn1 overexpression resulted in partial restoration of angiogenic differentiation ability (Fig. 1F). Flow cytometry was used to assess apoptosis. We found that circ-Astn1 overexpression suppressed HG-induced EPC apoptosis (Fig. 1G and H). Inflammatory factor analysis verified that HG pretreatment increased the secretion of IL-1β, TNF-α, and IL-6, whereas circ-Astn1 overexpression suppressed the secretion of IL-1β, TNF-α and IL-6 under HG conditions (Fig. 1I–K).

### miR-138-5p and SIRT1 Are Downstream Targets of Circ-Astn1

**Fig. 2:**
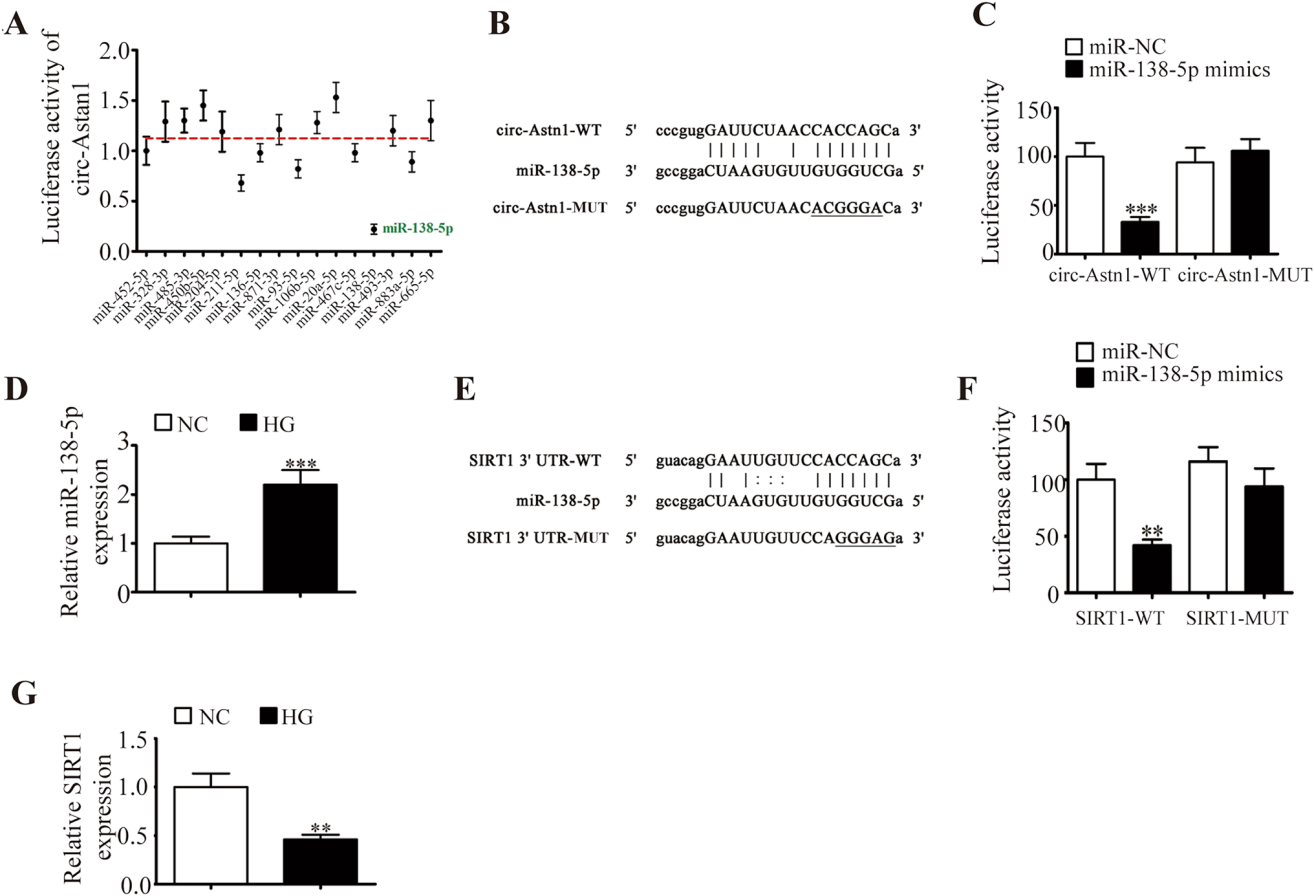
miR-138-5p and SIRT1: downstream targets of circ-Astn1. **A** Luciferase activity of circ-Astn1 in HEK293T cells transfected with various miRNA mimics that are putative binding sites for the circ-Astn1 sequence. Luciferase activity was normalized to Renilla luciferase activity. **B** Binding site predictions for miR-138-5p in circ-Astn1. MUT circ-Astn1 is shown. **C** Relative luciferase activity 2 days after HEK293T cell transfection with the miR-138-5p mimic/NC or circ-Astn1 MUT/WT. The results are expressed as the mean ± SD. **D** RT-qPCR analysis of miR-138-5p expression in HUVECs under NC or HG conditions. The results are expressed as the mean ± SD. **E** Binding site predictions for miR-138-5p within the SIRT1 3ʹUTR. MUT 3ʹ-UTR-SIRT1 is given. **F** Relative luciferase activity 2 days after HEK293T cell transfection with the miR-138-5p mimic/NC or 3ʹUTR-SIRT1 MUT/WT. The results are expressed as the mean ± SD. **G** RT-qPCR analysis of SIRT1 expression in EPCs under NC or HG conditions. The results are expressed as the mean ± SD. ^**^P < 0.01, ***P < 0.001 *vs.* NC

Recent studies have revealed that circRNAs regulate gene expression by sponging miRNAs [20]. Bioinformatics data predicted that circ-Astn1 might act with miRNAs such as miR-328-3p, miR-138-5p, miR-452-5p, miR-485-3p, miR-450b-5p, miR-204-5p, miR-211-5p, and miR-136-5p. First, we constructed a luciferase reporter vector with the circ-Astn1 sequence and transfected the vector along with various miRNA mimics into HEK293 cells. We found that only miR-138-5p led to decreased signal intensity, indicating that miR-138-5p is a downstream target of circ-Astn1 (Fig. 2A). Luciferase reporter analyses confirmed that miR-138-5p suppressed luciferase activity in WT cells but not in MUT cell lines (Fig. 2B and C), suggesting that miR-138-5p is a circ-Astn1 target. RT-qPCR confirmed that miR-138-5p expression increased in EPCs under HG conditions (Fig. 2D).

Bioinformatics analysis identified SIRT1 as a downstream target of miR-138-5p. To verify the correlation between miR-138-5p and SIRT1, the MUT or WT 3ʹUTR-SIRT1 sequences, such as the miR-138-5p binding sequence, were inserted into a luciferase reporter vector (Fig. 2E). Our laboratory transfected a luciferase reporter vector into HEK293T cells with or without the miR-138-5p mimic. The luciferase reporter results suggested that miR-138-5p suppressed luciferase activity in WT but not MUT cell lines (Fig. 2F), indicating that miR-138-5p is a SIRT1 target. RT-qPCR revealed that SIRT1 expression decreased in EPCs under HG conditions (Fig. 2G).

### SIRT1 Downregulation or miR-138-5p Overexpression Reversed the Protective Effect of Circ-Astn1 on HG-Induced EPC Dysfunction

**Fig. 3:**
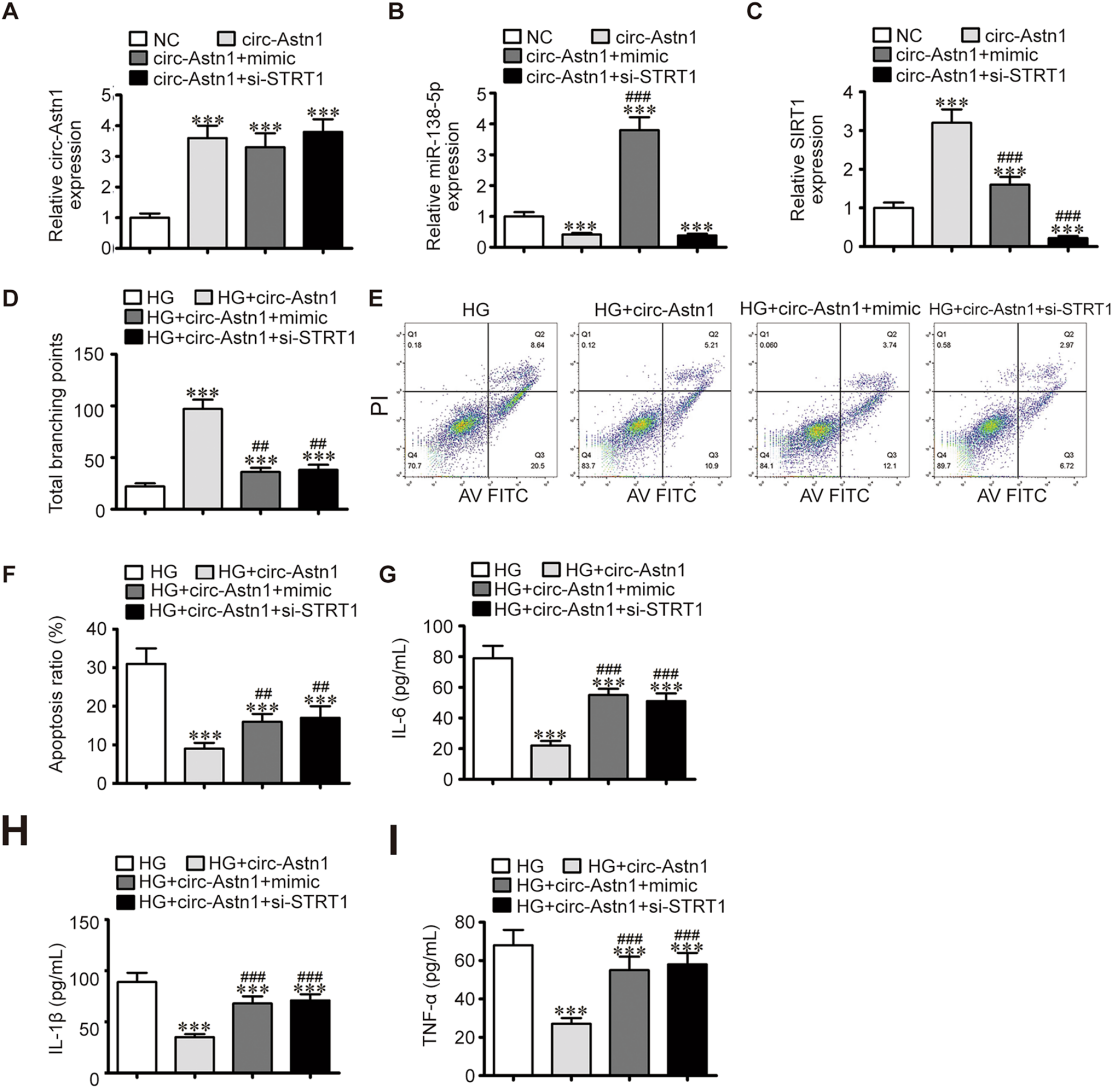
The protective effects of circ-Astn1 on HG-induced endothelial cell dysfunction reversed by SIRT1 overexpression or miR-138-5p suppression **A–C** RT-qPCR analysis expression of circ-Astn1 (**A**), miR-138-5p (**B**), and SIRT1 (**C**) in EPCs. The data are expressed as the mean ± SD. **D** The tube formation ability of HUVECs. The data are expressed as the mean ± SD. **E** and **F** The percentage of apoptotic EPCs determined by annexin-V/PI staining after 1 day under NC or HG conditions. The data are expressed as the mean ± SD. **G–I** The levels of the inflammatory cytokines IL-6 (**G**), IL-1β (**H**), and TNF-α (**I**) measured via ELISA. The data are expressed as the mean ± SD. ^***^P < 0.001 *vs.* NC, ^##^*P* < 0.01, ^###^*P* < 0.001 *vs*. circ-Astn1

RT-qPCR data revealed that circ-Astn1 expression was increased after circ-Astn1 overexpression vector transfection. However, the miR-138-5p mimic or SIRT1 downregulation had no effect on circ-Astn1 expression (Fig. 3A), indicating that miR-138-5p and SIRT1 are downstream targets of circ-Astn1. RT-qPCR analysis revealed that circ-Astn1 overexpression decreased miR-138-5p expression. However, SIRT1 silencing did not reverse the inhibitory effect of circ-Astn1 on miR-138-5p expression (Fig. 3B), suggesting that miR-138-5p is a downstream target of circ-Astn1. Our team discovered that circ-Astn1 overexpression increased SIRT1 expression, but upregulation of miR-138-5p reversed this effect. Following SIRT1 silencing, SIRT1 expression decreased significantly (Fig. 3C). Together, these data suggest that circ-Astn1 promotes SIRT1 expression via the sponging of miR-138-5p.

Under HG conditions, circ-Astn1 overexpression restored the angiogenic differentiation ability of EPCs, as evidenced by increased formation of capillary-like tube structures. Nevertheless, miR-138-5p overexpression or SIRT1 silencing inhibited angiogenic differentiation following circ-Astn1 overexpression (Fig. 3D). Flow cytometry analysis of apoptosis revealed that circ-Astn1 overexpression suppressed HG-induced EPC apoptosis, but miR-138-5p overexpression or SIRT1 silencing maintained HG-induced EPC apoptosis (Fig. 3E and F). Inflammatory factor analysis revealed that HG pretreatment increased the secretion of IL-1β, TNF-α and IL-6. However, miR-138-5p overexpression or SIRT1 silencing maintained TNF-α, IL-1β and IL-6 secretion under HG conditions (Fig. 3G–I).

### SIRT1 Overexpression Reversed miR-138-5p-induced Endothelial Cell Dysfunction under HG Conditions

**Fig. 4:**
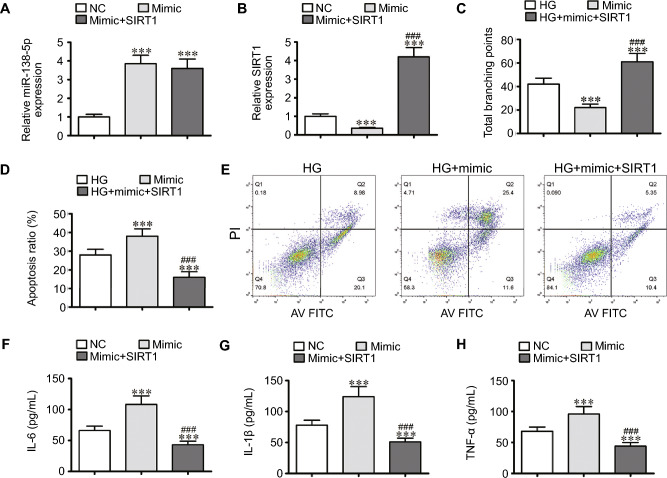
miR-138-5p-induced endothelial cell dysfunction under HG conditions reversed by SIRT1 overexpression. **A** and **B** RT-qPCR data showing the expression of miR-138-5p (**A**) and SIRT1 (**B**) in EPCs. The results are expressed as the mean ± SD. **C** EPCs tube formation ability. The results are expressed as the mean ± SD. **D** and **E** The percentage of apoptotic EPCs determined by annexin-V/PI staining after 1 day under NC or HG conditions. The results are expressed as the mean ± SD. **F–H** The levels of the inflammatory cytokines IL-6 (**F**), IL-1β (**G**), and TNF-α (**H**) measured via ELISA. The results are expressed as the mean ± SD. ^***^P < 0.001 *vs*. NC, ^###^*P* < 0.001 *vs*. mimic

RT‒qPCR data verified that miR-138-5p expression promoted miR-138-5p mimic transfection. Furthermore, treatment with the SIRT1 overexpression vector did not influence miR-138-5p expression (Fig. 4A), indicating that SIRT1 is downstream of miR-138-5p. The RT‒qPCR data suggested that miR-138-5p overexpression decreased SIRT1 expression. Following SIRT1 overexpression vector transfection, SIRT1 expression was significantly increased (Fig. 4B), suggesting that miR-138-5p decreased SIRT1 expression.

Under HG conditions, miR-138-5p overexpression further decreased the angiogenic differentiation ability of EPCs, as evidenced by the decrease in the number of capillary-like tube structures observed. However, SIRT1 overexpression restored angiogenic differentiation ability under HG conditions (Fig. 4C). Flow cytometric analysis verified that miR-138-5p overexpression increased HG-induced EPC apoptosis, but miR-138-5p overexpression suppressed HG-induced EPC apoptosis (Fig. 4D and E). Inflammatory factor analysis suggested that miR-138-5p overexpression enhanced TNF-α, L-1β and IL-6 secretion under HG conditions. Nevertheless, SIRT1 overexpression reversed the inhibitory effects of miR-138-5p overexpression on IL-6, TNF-α and IL-1β secretion (Fig. 4F–H).

### Circ-Astn1 Overexpression Promoted Skin Wound Healing through the Regulation of SIRT1

**Fig. 5:**
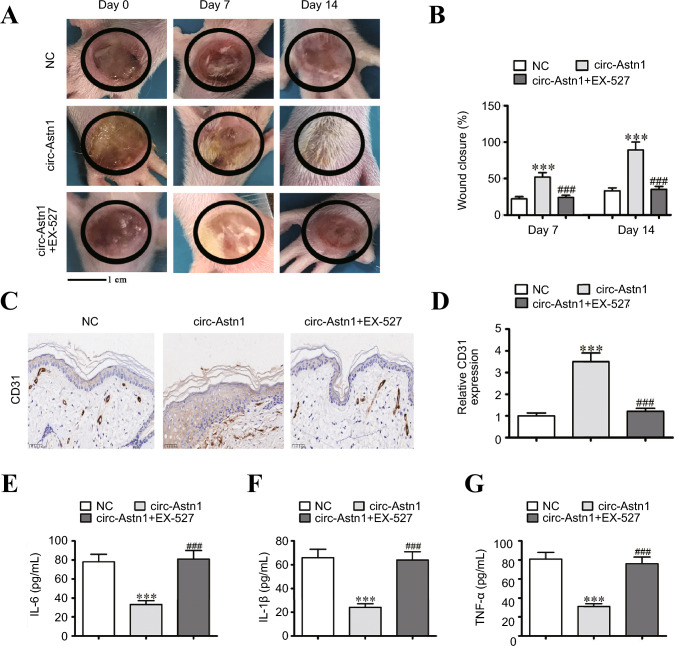
Skin wound healing promoted by circ-Astn1 overexpression via the regulation of SIRT1. **A** and **B** Images of skin wounds from db/db mice that received the circ-Astn1 overexpression lentiviral vector and combined treatment with the circ-Astn1 overexpression lentiviral vector and EX-527 (a SIRT1 inhibitor, 5 mg/kg/day); *n* = 6 mice in each group. The data are expressed as the mean ± SD. **C** and **D** Immunohistochemical analysis of angiogenesis in wound tissue via CD31 staining. The data are expressed as the mean ± SD. **E–G** The levels of the inflammatory cytokines IL-6 (**E**), IL-1β (**F**), and TNF-α (**G**) in the wound tissue were measured via ELISA. The results are expressed as the mean ± SD. ^***^P < 0.001 *vs*. NC,.^###^*P* < 0.001 *vs*. circ-Astn1

Next, we constructed a skin wound model in db/db mice. After a circ-Astn1 overexpression lentiviral vector was applied, wound healing was enhanced. However, when the circ-Astn1 overexpression vector was applied at 5 mg/kg/day in combination with the SIRT1 inhibitor EX-527, the wound healing ability was reduced (Fig. 5A and B). Immunohistochemical CD31 staining of the wound tissue revealed that circ-Astn1 overexpression accelerated angiogenesis, which was not observed after treatment with EX-527 (Fig. 5C and D). IL-1β, TNF-α and IL-6 levels in wound tissue were captured via ELISA, which revealed that circ-Astn1 overexpression decreased inflammatory cytokine expression, whereas SIRT1 inhibitor treatment promoted inflammatory cytokine expression (Fig. 5E–G). Together, these data suggest that circ-Astn1 overexpression promotes skin wound healing via the regulation of SIRT1.

## Discussions

Previous investigations have shown that chronic nonhealing wounds are a quite common diabetes complication. These wounds require integrative treatment strategies [21]. Disparate attempts have aimed to improve the diabetic wound healing process; however, ideal therapeutic strategies are limited. The present study revealed that circ-Astn1 expression decreased in both diabetic mice and EPCs under HG conditions. circ-Astn1 overexpression reversed HG-induced endothelial cell dysfunction, suggesting that circ-Astn1 plays a regulatory role in the pathogenesis of DM. Previous studies reported that circRNAs may function in DM development [22]. The circRNA hsa_circ_0084443 modulates keratinocyte proliferation and migration and was found to be upregulated in diabetic foot ulcers [23]. Serum and exosome hsa_circ_0057362 and hsa_circ_0000907 serve as new markers for early diabetic foot ulcer diagnosis [24]. Hsa_circ_0041795 contributes to HG-induced injury in the human retinal pigment epithelial cell line ARPE-19 via miR-646 sponging and activation of *VEGFC* [25]. However, the regulatory mechanism of circ-Astn1 in diabetic vascular dysfunction is still unclear.

To further identify downstream miRNAs that might interact with circ-Astn1, bioinformatics analysis was employed, and 16 candidate miRNAs were found to potentially be targeted by circ-Astn1. Luciferase reporter experiments confirmed that circ-Astn1 interacted with miR-138-5p and that circ-Astn1 upregulation reduced miR-138-5p expression. Our team verified that miR-138-5p expression increased in HG-induced EPCs, which is concordant with prior results [26]. A previous study reported that miR-138-5p significantly suppressed trophoblast migration and proliferation [27], and another study revealed that astragaloside-IV alleviates HG-induced ferroptosis in retinal pigment epithelial cells by disrupting the expression of miR-138-5p/Sirt1/Nrf2 [28]. Furthermore, miR-138 is utilized to distinguish diabetic patients from obese diabetic patients [29]. These findings suggest that miR-138-5p overexpression contributes to HG-induced endothelial cell dysfunction. In this context, our team hypothesized that circ-Astn1 expression would improve endothelial cell function through miR-138-5p sponging.

More results revealed that miR-138-5p interacted with the 3ʹUTR of SIRT1, which was verified with a luciferase reporter assay. Our team discovered that miR-138-5p expression was increased in HG-induced EPCs and decreased in HG-induced EPCs. The overexpression of circ-Astn1 promoted SIRT1 expression, but the overexpression of miR-138-5p reduced SIRT1 expression. SIRT1 silencing reversed the protective effect of circ-Astn1 on HG-induced endothelial cell function. SIRT1 overexpression restored endothelial cell function under HG conditions, as did miR-138-5p overexpression. Previous investigations revealed that SIRT1 activation leads to NF-κB downregulation as well as the suppression of inflammatory factors [30]. SIRT1 is elevated by naturally occurring antioxidants and anti-inflammatory compounds such as resveratrol, trans-δ-viniferin, and vitamin D [31]. Poly-ADP ribose polymerase (PARP)-1 inhibition protects diabetic hearts via activation of the SIRT1-PGC-1α axis [32]. Our study revealed that SIRT1 activation increased survival and maintained function through decreasing apoptosis and the expression of HG-induced inflammatory cytokines.

In conclusion, our data show that circ-Astn1 expression promotes survival and enhances endothelial cell function in EPCs, likely through miR-138-5p/SIRT1 signaling. Together, our findings suggest that circ-Astn1 is a promising diabetes diagnostic biomarker. Furthermore, our findings may facilitate expanded drug applications targeting circ-Astn1 and highlight a potential role for circ-Astn1 in diabetes treatment.

## Data Availability

The datasets used and/or analyzed during the current study are available from the corresponding author on reasonable request.

## References

[1] Cloete L. Diabetes mellitus: an overview of the types, symptoms, complications and management. Nurs Stand. 2022;37(1):61–66. 34708622 10.7748/ns.2021.e11709

[2] Darenskaya MA, Kolesnikova LI, Kolesnikov SI. Oxidative Stress: Pathogenetic Role in Diabetes Mellitus and Its Complications and Therapeutic Approaches to Correction. Bull Exp Biol Med. 2021;171(2):179–189. 34173093 10.1007/s10517-021-05191-7 PMC8233182

[3] Stachura A, Khanna I, Krysiak P, et al*.* Wound Healing Impairment in Type 2 Diabetes Model of Leptin-Deficient Mice-A Mechanistic Systematic Review. Int J Mol Sci. 2022;23(15):8621. 35955751 10.3390/ijms23158621 PMC9369324

[4] Hong YK, Chang YH, Lin YC, et al*.* Inflammation in Wound Healing and Pathological Scarring. Adv Wound Care (New Rochelle). 2023;12(5):288–300. 36541356 10.1089/wound.2021.0161

[5] Hassanshahi A, Moradzad M, Ghalamkari S, et al*.* Macrophage-Mediated Inflammation in Skin Wound Healing. Cells. 2022;11(19):2953. 36230913 10.3390/cells11192953 PMC9564023

[6] Okonkwo UA, DiPietro LA. Diabetes and Wound Angiogenesis. Int J Mol Sci. 2017;18(7),1419. 28671607 10.3390/ijms18071419 PMC5535911

[7] Shen J, Zhao X, Zhong Y, et al*.* Exosomal ncRNAs: The pivotal players in diabetic wound healing. Front Immunol. 2022;13:1005307. 36420273 10.3389/fimmu.2022.1005307 PMC9677725

[8] Meng Z, Zhou D, Gao Y, et al*.* miRNA delivery for skin wound healing. Adv Drug Deliv Rev. 2018;129:308–318. 29273517 10.1016/j.addr.2017.12.011

[9] Tang YB, Uwimana MMP, Zhu SQ, et al*.* Non-coding RNAs: Role in diabetic foot and wound healing. World J Diabetes. 2022;13(12):1001–1013. 36578864 10.4239/wjd.v13.i12.1001 PMC9791568

[10] Li X, Li N, Li B, et al*.* Noncoding RNAs and RNA-binding proteins in diabetic wound healing. Bioorg Med Chem Lett. 2021;50:128311. 34438011 10.1016/j.bmcl.2021.128311

[11] Herter EK, Xu Landen N. Non-Coding RNAs: New Players in Skin Wound Healing. Adv Wound Care (New Rochelle). 2017;6(3):93–107. 28289554 10.1089/wound.2016.0711 PMC5346954

[12] Ross K. MiR equal than others: MicroRNA enhancement for cutaneous wound healing. J Cell Physiol. 2021;236(12):8050–8059. 34160067 10.1002/jcp.30485

[13] Zhang Y, Tao K, Ding L, et al*.* Assessing biomarkers for post-surgical wound healing: A meta-analysis of exosome-based CircRNA in breast cancer recovery. Int Wound J. 2024;21(2):e14723. 38379248 10.1111/iwj.14723 PMC10830351

[14] Chen J, Wu Y, Luo X, et al*.* Circular RNA circRHOBTB3 represses metastasis by regulating the HuR-mediated mRNA stability of PTBP1 in colorectal cancer. Theranostics. 2021;11(15):7507–7526. 34158864 10.7150/thno.59546 PMC8210600

[15] Tong KL, Tan KE, Lim YY, et al*.* CircRNA-miRNA interactions in atherogenesis. Mol Cell Biochem. 2022;477(12):2703–2733. 35604519 10.1007/s11010-022-04455-8

[16] Li Y, Cheng T, Wan C, et al*.* circRNA_0084043 contributes to the progression of diabetic retinopathy via sponging miR-140-3p and inducing TGFA gene expression in retinal pigment epithelial cells. Gene. 2020;747:144653. 32259630 10.1016/j.gene.2020.144653

[17] Liu C, Yao MD, Li CP, et al*.* Silencing Of Circular RNA-ZNF609 Ameliorates Vascular Endothelial Dysfunction. Theranostics. 2017;7(11):2863–2877. 28824721 10.7150/thno.19353 PMC5562221

[18] An T, Zhang J, Ma Y, et al*.* Relationships of Non-coding RNA with diabetes and depression. Sci Rep. 2019;9(1):10707. 31341180 10.1038/s41598-019-47077-9 PMC6656886

[19] Huang S, Xu M, Li M, et al*.* The Expression of Circ-Astn1 Inhibits High Glucose Induced Endothelial Progenitor Cell Dysfunction by Activating Autophagy. Endocr Res. 2024;49(4):213–222. 38867680 10.1080/07435800.2024.2365887

[20] Panda AC. Circular RNAs Act as miRNA Sponges. Adv Exp Med Biol. 2018;1087:67–79. 30259358 10.1007/978-981-13-1426-1_6

[21] Chen CY, Rao SS, Ren L, et al*.* Exosomal DMBT1 from human urine-derived stem cells facilitates diabetic wound repair by promoting angiogenesis. Theranostics. 2018;8(6):1607–1623. 29556344 10.7150/thno.22958 PMC5858170

[22] Yang ZG, Awan FM, Du WW, et al*.* The Circular RNA Interacts with STAT3, Increasing Its Nuclear Translocation and Wound Repair by Modulating Dnmt3a and miR-17 Function. Mol Ther. 2017;25(9):2062–2074. 28676341 10.1016/j.ymthe.2017.05.022 PMC5589065

[23] Wang A, Toma MA, Ma J, et al*.* Circular RNA hsa_circ_0084443 Is Upregulated in Diabetic Foot Ulcer and Modulates Keratinocyte Migration and Proliferation. Adv Wound Care (New Rochelle). 2020;9(4):145–160. 32117579 10.1089/wound.2019.0956 PMC7047102

[24] Chen ZJ, Shi XJ, Fu LJ, et al*.* Serum and exosomal hsa_circ_0000907 and hsa_circ_0057362 as novel biomarkers in the early diagnosis of diabetic foot ulcer. Eur Rev Med Pharmacol Sci. 2020;24(15):8117–8126. 32767340 10.26355/eurrev_202008_22498

[25] Sun H, Kang X. hsa_circ_0041795 contributes to human retinal pigment epithelial cells (ARPE 19) injury induced by high glucose via sponging miR-646 and activating VEGFC. Gene. 2020;747:144654. 32259632 10.1016/j.gene.2020.144654

[26] Luan B, Sun C. MiR-138-5p affects insulin resistance to regulate type 2 diabetes progression through inducing autophagy in HepG2 cells by regulating SIRT1. Nutr Res. 2018;59:90–98. 30442237 10.1016/j.nutres.2018.05.001

[27] Ding R, Guo F, Zhang Y, et al*.* Integrated Transcriptome Sequencing Analysis Reveals Role of miR-138-5p/ TBL1X in Placenta from Gestational Diabetes Mellitus. Cell Physiol Biochem. 2018;51(2):630–646. 30463081 10.1159/000495319

[28] Tang X, Li X, Zhang D, et al. Astragaloside-IV alleviates high glucose-induced ferroptosis in retinal pigment epithelial cells by disrupting the expression of miR-138-5p/Sirt1/Nrf2. Bioengineered. 2022;13(4):8240–8254. 35302431 10.1080/21655979.2022.2049471 PMC9162003

[29] Pescador N, Perez-Barba M, Ibarra JM, et al. Serum circulating microRNA profiling for identification of potential type 2 diabetes and obesity biomarkers. PLoS One. 2013;8(10):e77251. 24204780 10.1371/journal.pone.0077251 PMC3817315

[30] Karbasforooshan H, Karimi G. The role of SIRT1 in diabetic retinopathy. Biomed Pharmacother. 2018;97:190–194. 29091865 10.1016/j.biopha.2017.10.075

[31] Strycharz J, Rygielska Z, Swiderska E, et al*.* SIRT1 as a Therapeutic Target in Diabetic Complications. Curr Med Chem. 2018;25(9):1002–1035. 29110598 10.2174/0929867324666171107103114

[32] Waldman M, Nudelman V, Shainberg A, et al*.* PARP-1 inhibition protects the diabetic heart through activation of SIRT1-PGC-1alpha axis. Exp Cell Res. 2018;373(1-2):112–118. 30359575 10.1016/j.yexcr.2018.10.003

